# The estimation of patient-specific cardiac diastolic functions from clinical measurements

**DOI:** 10.1016/j.media.2012.08.001

**Published:** 2012-10-16

**Authors:** Jiahe Xi, Pablo Lamata, Steven Niederer, Sander Land, Wenzhe Shi, Xiahai Zhuang, Sebastien Ourselin, Simon G. Duckett, Anoop K. Shetty, C. Aldo Rinaldi, Daniel Rueckert, Reza Razavi, Nic P. Smith

**Affiliations:** a Department of Computer Science, University of Oxford, United Kingdom; b Department of Biomedical Engineering, Kings College London, Kings Health Partners, St. Thomas Hospital, London SE1 7EH, United Kingdom; c Department of Computing, Imperial College London, United Kingdom; d Centre for Medical Image Computing, University College London, United Kingdom

**Keywords:** Constitutive material parameter estimation, Left ventricular (LV) mechanics, Diastolic heart failure

## Abstract

An unresolved issue in patients with diastolic dysfunction is that the estimation of myocardial stiffness cannot be decoupled from diastolic residual active tension (AT) because of the impaired ventricular relaxation during diastole. To address this problem, this paper presents a method for estimating diastolic mechanical parameters of the left ventricle (LV) from cine and tagged MRI measurements and LV cavity pressure recordings, separating the passive myocardial constitutive properties and diastolic residual AT. Dynamic *C*_1_-continuous meshes are automatically built from the anatomy and deformation captured from dynamic MRI sequences. Diastolic deformation is simulated using a mechanical model that combines passive and active material properties. The problem of non-uniqueness of constitutive parameter estimation using the well known Guccione law is characterized by reformulation of this law. Using this reformulated form, and by constraining the constitutive parameters to be constant across time points during diastole, we separate the effects of passive constitutive properties and the residual AT during diastolic relaxation. Finally, the method is applied to two clinical cases and one control, demonstrating that increased residual AT during diastole provides a potential novel index for delineating healthy and pathological cases.

## 1 Introduction

The quantification of diastolic dysfunction is vital for the diagnosis and assessment of heart disease, enabling improved selection and treatment of individuals with pathological myocardial mechanics for further therapy (Nagel and Schuster, 2010). Patient-specific cardiac models, parameterized from clinical measurements on an individual basis, provide a powerful approach for this purpose (Smith et al., 2011). Accordingly, model-based parameter estimation from clinical measurements of cardiac function has been an active research area.

Parameters in organ-level cardiac mechanical models can be broadly classified as passive and active. Typically within computational models, passive constitutive parameters have been used to characterize the diastolic function, and with the addition of active contraction models simulate systole (Nash and Hunter, 2000; Nordsletten et al., 2011). Various frameworks and methods have been proposed to estimate these parameters (Sermesant et al., 2006, 2012; Chabiniok et al., 2011; Delingette et al., 2012; Moireau and Chapelle, 2011; Wang et al., 2009, 2010). In Sermesant et al. (2006), a variational data assimilation method was developed to estimate the contractility parameters of an electromechanical model from clinical cine MRI. Focusing on passive parameters, Wang et al. (2009) have described a workflow to estimate the Guccione constitutive parameters using high-resolution MRI data acquired from a canine heart. An approach which these authors further extended in Wang et al. (2010) to estimate the active tension (AT) during the isovolumetric contraction, systole and isovolumetric relaxation using the constitutive parameters pre-estimated during diastole.

However, an unsolved problem in patients with diastolic dysfunction is that the estimation of myocardial stiffness cannot be decoupled from impaired ventricular relaxation (one of the lusitropic abnormalities commonly present in heart failure, Katz, 2010). For this reason, the development of methods which can robustly estimate both the stiffness and residual AT during diastole would have significant potential for application within clinical cardiology.

The focus of this study is to address this issue directly through the inclusion and estimation of an AT term during diastole. Specifically, built upon the parameter estimation framework for passive constitutive properties in our previous work (Xi et al., 2011b), we propose an approach to further estimate the residual AT during diastole to directly characterize the delayed relaxation often present in heart failure patients. We first undertake the necessary step for our estimation problem of reformulating the constitutive law to reveal and address the issue of the non-uniqueness of material parameters. Using this reformulated form, we then introduce an AT term in our mechanical model and estimate the residual AT in early diastole. Finally we apply this methodology to clinical cases with pressure, cine and tagged MRI measurements, with the results showing that estimated myocardial stiffness and residual AT appear to be promising candidates to delineate healthy and pathological patient cases.

## 2 Materials and methods

**Fig. 1:**
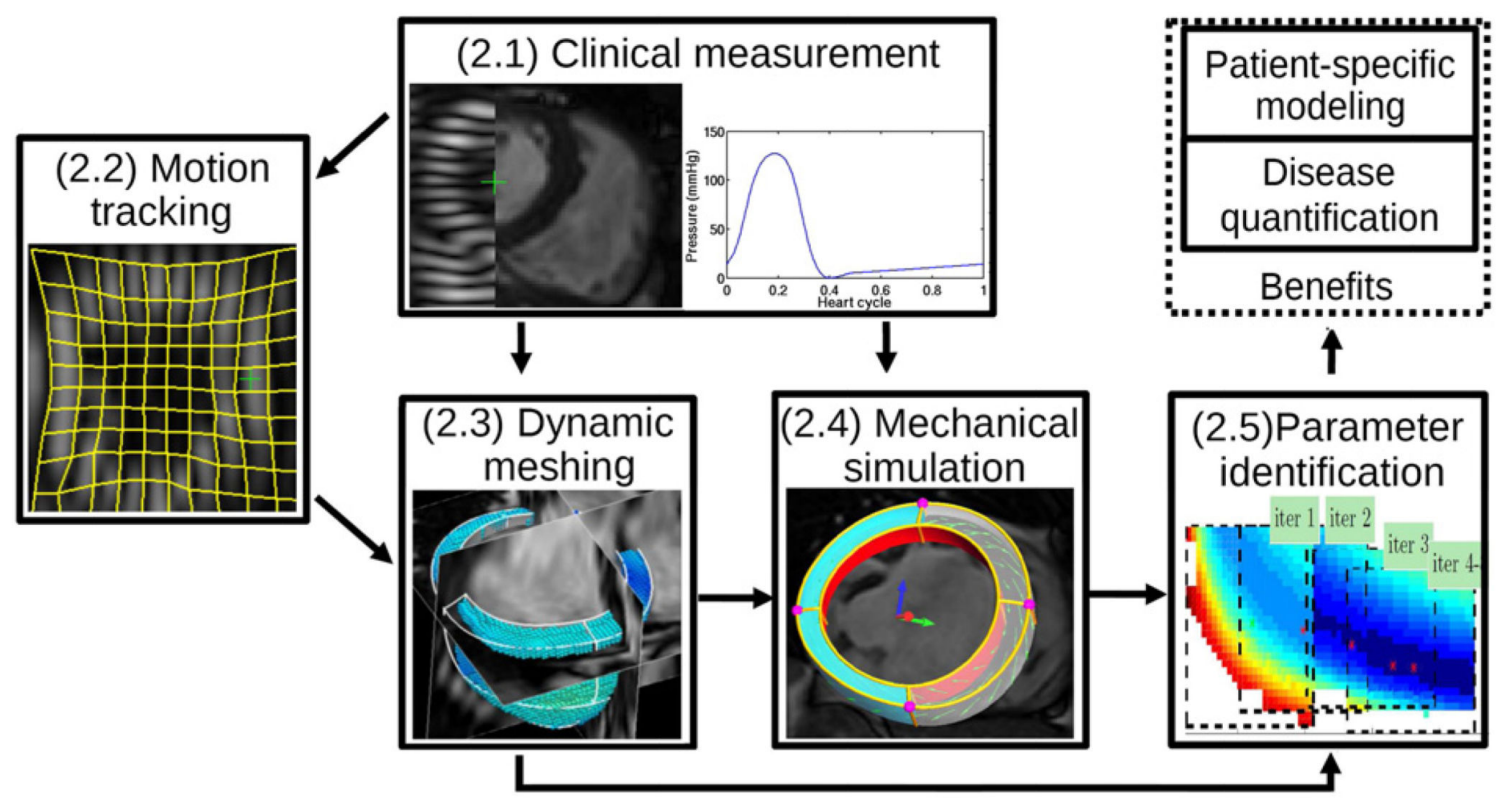
Workflow of proposed data assimilation framework for patient-specific parameter estimation. The text labels correspond to the section number in this paper.

The constitutive parameters and residual AT are identified by comparing simulated diastolic inflation to a set of observed deformations extracted from combined cine and 3D tagged MRI data. Specifically, passive filling of the human left ventricle (LV) is simulated using patient-specific geometry, with the loading condition determined from LV cavity pressure recordings. The geometry are obtained with an automatic dynamic meshing process, which captures the LV anatomy using one frame of the cine MRI data. Fig. 1 schematically illustrates this complete process, where the numbered labels correspond to the subsequent sections in this paper.

### 2.1 Clinical measurements

The data used in this study were acquired from two patients selected for Cardiac Resynchronization Therapy (CRT) in St Thomas’ Hospital, London and one healthy subject for control. This study conforms to the principles outlined in the Declaration of Helsinki and was carried out as part of a local ethics committee-approved protocol with informed consent obtained from the patients. Patient case 1 is a 74-year-old female with NYHA Class II heart failure despite optimal medical treatment. There was significant LV systolic dysfunction with an LV ejection fraction of 16% and QRS duration of 168 ms. The LV is significantly dilated with an end systolic volume (ESV) of 335 ml. Patient case 2, a 78-year-old male, has the same disease classification as case 1, with ejection fraction of 17% and an ESV of 186 ml. The control case used in this study is a healthy 36-year-old male.

**Fig. 2:**
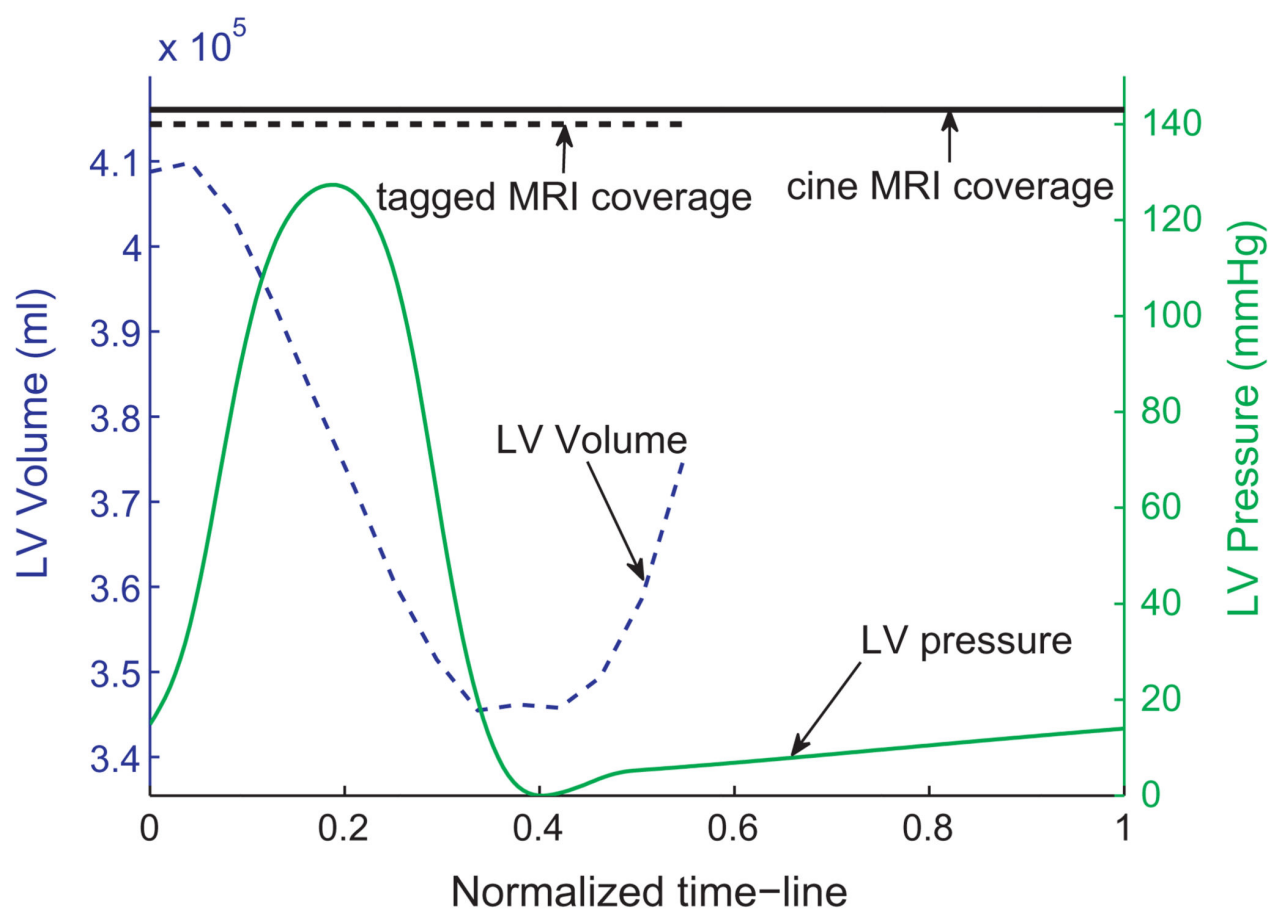
The coverage of cine and tagged MRI, pressure transient, and derived volume transient for patient case 1. The *x*-axis is the normalized heart cycle (R–R interval). The top horizontal line shows the coverage of the 29 frames of cine MRI and the 23 frames of tagged MRI. The beginning of diastole is at the frame 18 of the tagged MRI sequence, and thus five frames at early diastole are available while the frame of end-diastole is assumed to be synchronous to the R wave. The volume transient is calculated as the LV cavity volume of the fitted FM meshes (described in Section 2.3.2). The pressure transient is the averaged value recorded over multiple heart cycles.

For each of these three data set, cardiac deformation is characterized by spatially aligned cine MRI (29 frames per heart cycle, short-axis view, vowel size 1.3 × 1.3 × 10 mm) and 3D tagged MRI (23 frames per heart cycle, vowel size 0.96 × 0.96 × 0.96 mm, tagging line width ~5 vowels). The LV cavity pressure transient is obtained from the cardiac catheterization procedure (separately from the MR scan), when the rate of change of LV pressure is measured. Fig. 2 summarizes the data set of patient case 1. The diastolic cavity pressure for the healthy case is taken by digitalizing the data of a typical pressure profile (Klabunde, 2005, Chapter 4, p. 62). End diastolic pressure is 1.47 kPa for the control case, while cases 1 and 2 are 1.93 and 1.69 kPa respectively.

### 2.2 Myocardial motion tracking

Critical information for the guidance of mechanical parameter estimation are the 3-dimensional displacements of *N* tracked myocardial points, or a time series of the Lagrangian displacement vectors from frame *j* to frame *i*{*_j_**Z**_i_* ∈ ℝ^3*N*^ | *i* = 1, 2, …, *T*} where *T* is the number of MRI frames. The automatic extraction of these displacements from the combined short-axis cine and 3D tagged MRI is performed with the Image Registration Toolkit,^1^ which uses a non-rigid registration method based on free-form deformations developed by Rueckert et al. (1999) and extended to the cardiac MRI motion tracking by Chandrashekara et al. (2004). Temporal alignment is achieved by interpolating cine MRI at the time points of tagged MRI. The spatial alignment of cine and tagged MRI is done by rigid registration between cine MRI and detagged tagged MRI, and the tagging line is removed in Fourier space. The aligned short-axis cine and tagged MRI provides information on the ventricular radial movement while the long-axis tagged MRI characterizes the apex-to-base movement. This complete process of motion tracking using combined information from cine and tagged MRI is detailed by Shi et al. (2012), which shows the relative registration error compared to manually tracked landmarks is less than 15% of the cardiac displacement throughout the cardiac cycle.

### 2.3 Dynamic mesh personalization

#### 2.3.1 Geometric model construction

Based on clinical segmentation of the end-diastolic (ED) frame of cine MRI, the LV mechanical mesh at ED is built with cubic-Hermite (CH) elements using methods developed by Lamata et al. (2011) (see Fig. 4). This CH mesh, with nodal positions and derivatives as degrees of freedom (DOF), provides a *C*^1^-continuous representation of the geometry. The fiber field, representing the dominant orientation of tissue microstructure within the LV, is embedded in the geometric model with transmural heterogeneity (±60° as shown in Fig. 4), based on the data of Usyk et al. (2000). The fiber field values are stored as angles of fiber at each node and interpolated within the material space of the finite element mesh using tri-linear basis functions.

#### 2.3.2 Geometric model propagation

Given the constructed geometrical model at end-diastole and the 4D myocardial displacement field from Section 2.2, a simple technique is employed to propagate this geometrical model to the time points of the displacement field. Specifically, given an initial mesh fitted to the anatomical data at a time point ***U**_j_* (in our specific case this is at ED) and a time-series of *N* ~ 6000, regular grid points inside the LV myocardium, 3*N* ≫ 8*M* material points’ displacement *_j_**Z**_i_* ∈ ℝ^3*N*^ (with the size of ~18,000) from time point *j* to *i*, we find, for each of the time points *i* (*i* = 1, 2, …, *T*), the nodal positions and derivatives ***U**_i_* (or DOF vector, with the size of ~1872) that define a *M*-node cubic-Hermite mesh. This mesh is found by minimizing the error in the mesh approximation to the observed positions of data points, formulated as a standard linear least-squared minimization problem.

This mesh approximation error *e_i_* is defined as the *L*_2_ norm of the residual vector – the difference between the observed positions ***z**_i_* of material points at time point *i*

(1) $${\text{z}}_{i}={\text{H}}_{\xi }{\text{U}}_{j}{+}_{j}{\text{Z}}_{i}$$

and the embedded positions ***y***_i_ in the fitted mesh

(2) $${\text{y}}_{i}={\text{H}}_{\xi }{\text{U}}_{i},$$

i.e.,

(3) $$e_{i}={\left\|{\text{z}}_{i}-{\text{y}}_{i}\right\|}_{{𝕃}^{2}}={\left\|{\text{H}}_{\xi }{\text{U}}_{j}{+}_{j}{\text{Z}}_{i}-{\text{H}}_{\xi }{\text{U}}_{i}\right\|}_{{𝕃}^{2}},$$

where ***H***_ξ_ ∈ 𝕄^3*N*×8*M*^ is the shape matrix related to the CH basis functions (Smith et al., 2004), and ***H****_ξ_****U****_i_* is spatial coordinates of the local *ξ*-coordinates embedded in the mesh ***U**_i_*. Solving the standard linear weighted least-square minimization problem described in Eq. (3), we obtain the DOF vectors of new meshes {***U**_i_*} given by

(4) $${\text{U}}_{i}={\left({\text{H}}_{\xi }^{T}{\text{H}}_{\xi }\right)}^{-1}{\text{H}}_{\xi }^{T}\left({\text{H}}_{\xi }{\text{U}}_{j}{+}_{j}{\text{Z}}_{i}\right).$$

### 2.4 LV mechanical model

**Fig. 3:**
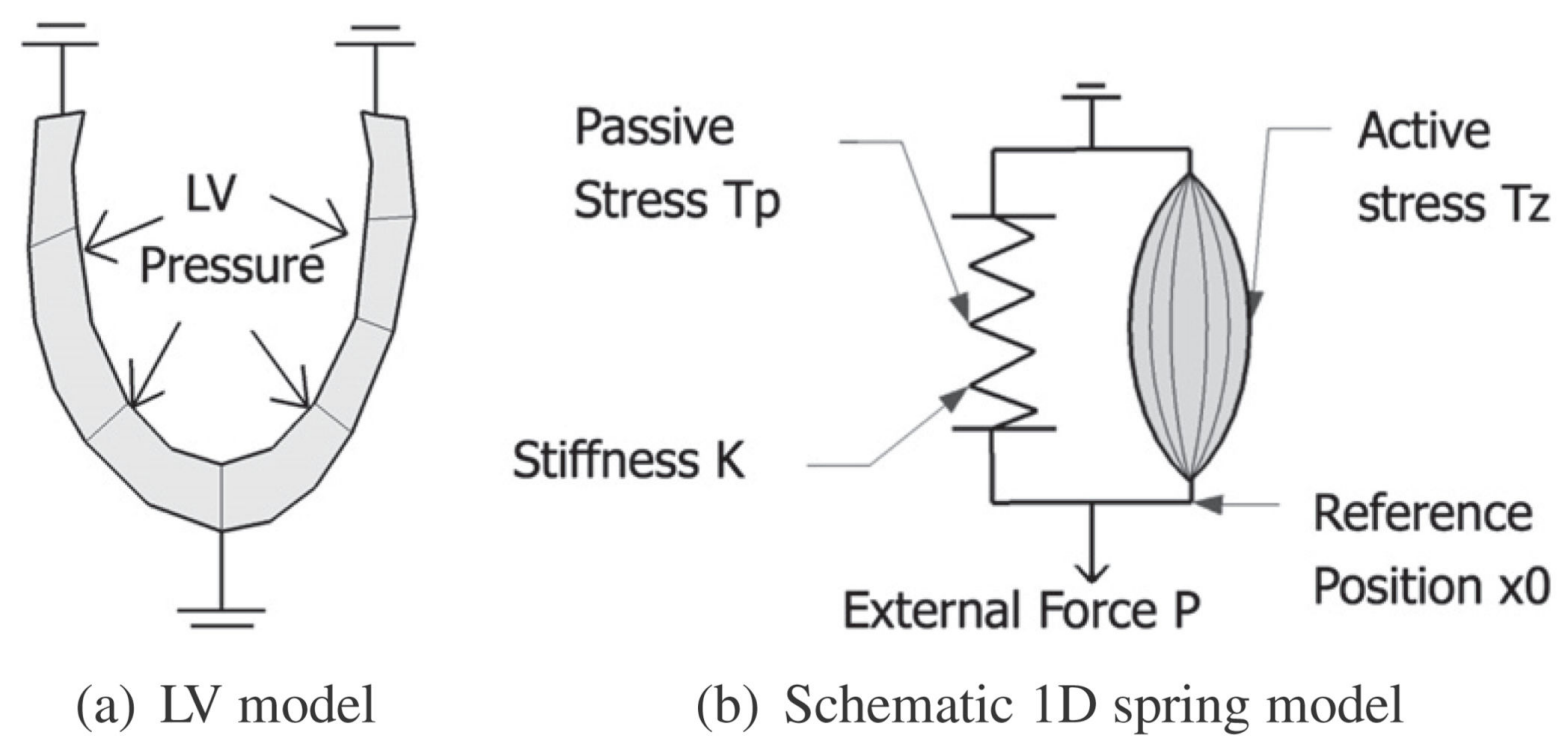
Schematic illustration of LV mechanical model. As illustrated by the schematic 1D spring model in subplot (b), the deformation of the spring (analogous to the deformation of LV myocardium in subplot (a)) is driven by two factors – the external force (analogous to the LV cavity pressure) and the active stress (analogous to the active tension developed by the contraction of the myocardial fiber).

The passive diastolic filling phase of the cardiac cycle is simulated by inflating the unloaded LV model up to cavity pressures corresponding to each frame of tagged MRI, as schematically illustrated by Fig. 3a. Deformation is then simulated using the standard finite deformation theory. The finite element method (FEM) is utilized to solve the stress equilibrium governing equation, with the measured LV cavity pressure being applied as the loading condition on the endocardium (Nash and Hunter, 2000; Nordsletten et al., 2011). The stress equilibrium governing equation is derived from the laws of conservation of mass and momentum, and the principle of virtual work. As demonstrated in Appendix B.1, our preliminary results necessitate an active tension term to be included in our mechanical model, to account for the residual tension generated by the contraction of myofibers. This active component is illustrated schematically by Fig. 3b, in which the 1D spring is not only stretched by the external force *P*, but also contracted by the active component parallel to the spring. The deformation of the spring, for this simple 1D case, is analogous to the deformation of the LV myocardium.

Our LV mechanical model, denoted by an operator 𝕄, determines the deformed position ***x***_i_ of material points whose initial positions at unloaded state are ***x***_0_. In addition to the unloaded state, the model operator also takes as its inputs ***C***∈ ℝ^4^ (constitutive parameters characterizing the stiffness of myocardium), *P_i_* (LV cavity pressure at time point i), *T_z_* (*i*) (residual AT), ${\text{y}}_{i}^{B}$ (displacement boundary conditions based on the observed displacements ***y**_i_*). That is,

(5) $${\text{x}}_{i}=𝕄\left({\text{x}}_{0},\text{C},P_{i},T_{z}\left(i\right),{\text{y}}_{i}^{B}\right).$$

Each of these inputs are further explained in the following subsections. The backward mechanical model (or deflation model), denoted by the inverse operator 𝕄^−1^, takes the deformed position ***x***_i_ as its input and retrieves the unloaded initial position ***x***_0_ (Rajagopal et al., 2008).

(6) $${\text{x}}_{0}={\text{𝕄}}^{-1}\left({\text{x}}_{i},\text{C},P_{i},T_{z}\left(i\right),{\text{y}}_{i}^{B}\right).$$

#### 2.4.1 Boundary conditions

**Fig. 4:**
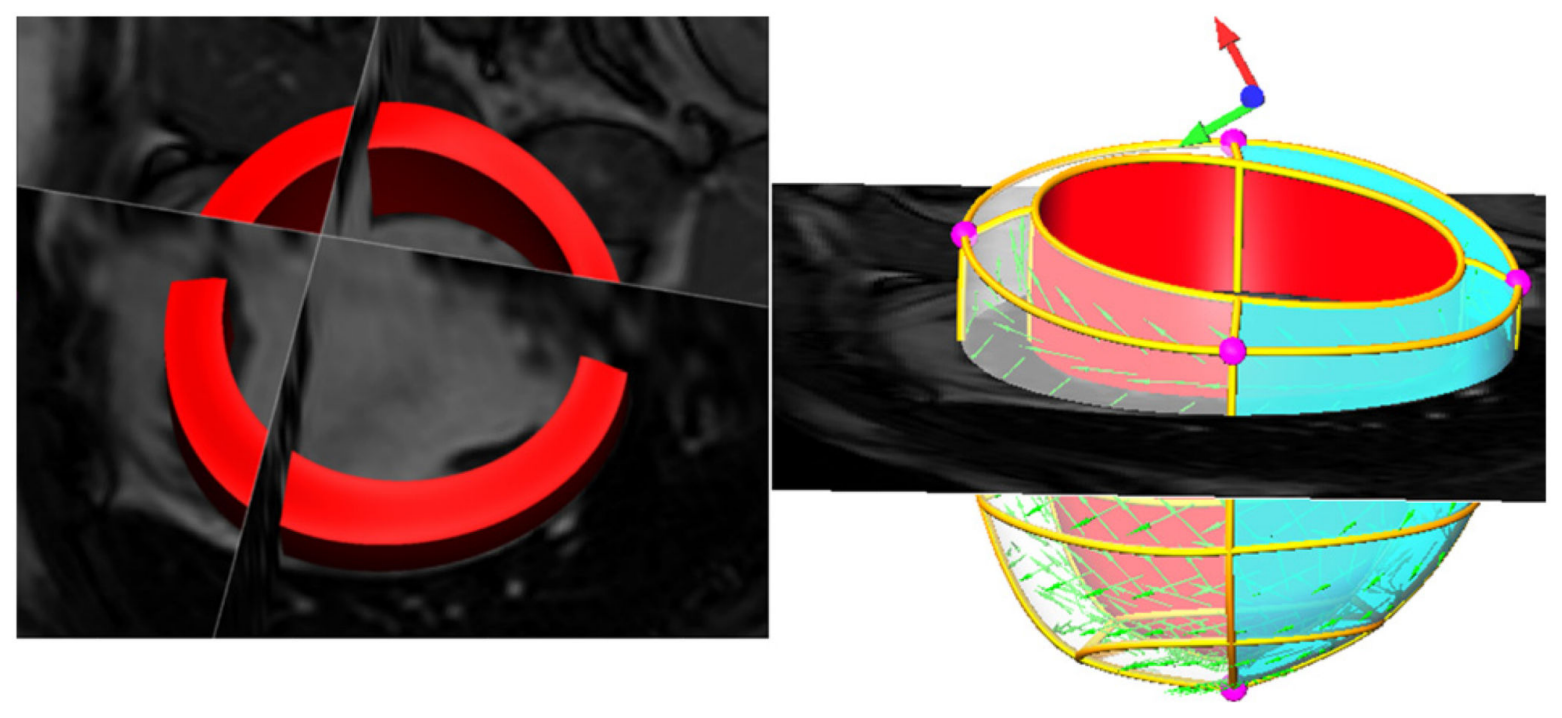
LV volumetric segmentation of the end-diastolic cine MRI (left) and geometric model built with fiber vectors embedded (right). In the geometric model, displacement boundary conditions are prescribed on the purple nodal points (four at the base plane and one in the apex). Only the movements of the free wall region (green area) are compared with the measurements in the parameter fitting process, since the model does not include the RV. (For interpretation of the references to color in this figure legend, the reader is referred to the web version of this article.)

The model developed in this study only represents the LV, and does not include representations of the right ventricle (RV), great vessels, pericardium and organs around the heart. Thus the effects of these structures on the LV mechanics are not explicitly modeled. To account for these physical constraints on the heart, we prescribe the kinematic movement of the LV model at its base plane and apex node (Fig. 4) to match the displacements extracted from the tagged MRI. This kind of patient-specific boundary conditions enable us to better compare our simulated meshes to the fitted meshes. Additionally, in order to minimize the influence of RV pressure on parameter estimation, only the movements of the free wall region (green area in Fig. 4) are compared with the measurements in the parameter fitting process.

#### 2.4.2 Constitutive parameters of the myocardium

Consistent with existing literature and based on the experimental results of uniaxial and biaxial tests in isolated cardiac muscle (Fung, 1981; Yin et al., 1987), the myocardium in this study is modeled as a transversely isotropic material with preferred directions that vary transmurally. We chose the widely employed 4-parameter Guccione law (Guccione et al., 1991) to balance the feasibility of estimating parameters with the ability to accurately account for the non-linear mechanical properties that result from the myocardial laminar structure. The Guccione strain-energy function *W* is defined as

(7) $$W=\underline{C_{1}}\left(e^{Q}-1\right),$$

(8) $$Q=\underline{C_{2}}E_{\text{ff}}^{2}+\underline{C_{3}}\left(E_{\text{ss}}^{2}+E_{\text{nn}}^{2}+2E_{\text{sn}}^{2}\right)+\underline{C_{4}}\left(2E_{\text{fs}}^{2}+2E_{\text{fn}}^{2}\right),$$

where *C*_1_, *C*_2_, *C*_3_ and *C*_4_ are the constitutive parameters to be estimated, *E_ff_*, *E_ss_* and *E_nn_* are the Green–Lagrange strains in fiber (*f*), sheet (*s*) and sheet normal (*n*) directions, and *E_sn_*, *E_fn_* and *E_fs_* are the Green–Lagrange shear strains in the *fs, fn* and *fs* planes. The *f, s* and *n* directions correspond to the fiber axes aligned with the microstructure of the myocardium.

#### 2.4.3 Reformulation of the Guccione law

As outlined in the introduction, in our previous study (Xi et al., 2011b), we identified that the difficulty with the Guccione formulation in the context of parameter estimation is that multiple parameter sets are able to reproduce similar end-diastolic deformation states. To clarify this issue further, we can reformulate the constitutive parameters by introducing *α* and *r*_2_–*r*_4_ as

(9) $$\underline{C_{1}}=C_{1,}$$

(10) $$\underline{\alpha }=C_{2}+C_{3}+C_{4},$$

(11) $$r_{2}=\frac{C_{2}}{\alpha }=1-r_{3}-r_{4},$$

(12) $$\underline{r_{3}}=\frac{C_{3}}{\alpha },$$

(13) $$\underline{r_{4}}=\frac{C_{4}}{\alpha },$$

where *α* and *r*_2_–*r*_4_ (non-negative) are the scale factor and anisotropies of *C*_2_–*C*_4_, respectively. The underlined parameters (*C*_1_, *α*, *r*_3_) and *r*_4_ on the left side of the above equations are those to be actually estimated in the following section. As we outline below, this reformulation uncouples *C*_1_–*C*_4_ into *C*_1_-*α* (homogeneous stiffness scale) and *r*_3_–*r*_4_ (anisotropy stiffness ratios), which clearly reveals the parameter correlation of the original Guccione’s law in the *C*_1_-*α* space. The motivation for using this formulated version of the law is that *C*_1_-*α* assists in the interpretation of parameter estimation results in terms of myocardial homogeneous stiffness, and *r*_2_ indicates the relative stiffness along the myofiber compared to other material directions. This coupling relationship was explained in detail in our previous work (Xi et al., 2011b). For completeness, this explanation is also summarized in Appendix A.1, including the plots of optimization objective function with respect to *C*_1_-*α* and *r*_3_–*r*_4_.

#### 2.4.4 Active tension model

In literature, the diastolic cardiac mechanics is usually modeled as pure passive inflation (e.g., Wang et al., 2009). That is, the myocardial stress is assumed to be the passive stress, caused by deformation of the elastic (incompressible) myocardial material.

(14) $$\text{T}=\frac{\partial W}{\partial \text{E}}+p{\text{C}}^{-1},$$

where ***T*** is the second Piola–Kirchhoff stress tensors, *W* is the strain energy function, and ***E*** is the Green–Lagrangian strain tensor in the local material directions. *p**C***^−1^ is the hydrostatic stress tensor because of the incompressible nature of the tissue, while $\frac{\partial W}{\partial \text{E}}$ is the deviatoric stress tensor due to the distortion of the tissue.

However, in our study, we found that the pure passive mechanical model could not fully explain the deformation presented in the early diastole (as demonstrated in Appendix B.1). To account for this discrepancy, we introduce a compensatory AT term along the fiber direction to the model.

(15) $$\text{T}=\underset{\text{deviatoric}\;\text{stress}\;\text{tensor}}{\underset{︸}{\frac{\partial W}{\partial \text{E}}}}+\underset{\text{hydrostatic}\;\text{stress}\;\text{tensor}}{\underset{︸}{p{\text{C}}^{-1}}}+\underset{\text{active}\;\text{stress}\;\text{tensor}}{\underset{︸}{\left(\begin{array}{ccc} T_{a} & 0 & 0\\ 0 & 0 & 0\\ 0 & 0 & 0 \end{array}\right)}},$$

where the length-dependent AT *T_a_*, as explained in the HMT model (Hunter et al., 1998; Nash, 1998; Wang et al., 2010), is defined by

(16) $$T_{a}=T_{\text{z}}\left(1+\beta \left(\sqrt{2E_{\text{ff}}+1}-1\right)\right),\;T_{\text{z}}=T_{\text{ref}}\cdot z.$$

In the above equation, *T_a_* is the length-dependent AT along fiber direction, *T*_ref_ is the maximum homogeneous reference tension, *T*_ref_ · *z* is the tension developed at activation level *z*(0 ⩽ *z* ⩽ 1), constant *β* is the coefficient for the linear length dependence of AT, *E*_ff_ is the Lagrangian–Green strain along fiber direction, and $\sqrt{2E_{\text{ff}}+1}-1$ is the extension ratio.

The activation level *z* during diastole, which can be obtained from electrical activation models such as monodomain or biodomain models (Smith et al., 2004), is assumed to be spatially homogeneous over the LV in our study. The combined *T*_ref_ · *z* term (renamed as *T_z_*, and referred as the “AT term” or “AT parameter” from now on) is estimated at each time point of diastolic MR images, using a pre-estimated constant passive material parameter set (explained in detail below). At the end-diastole, the heart is assumed to be completely relaxed (i.e., no residual AT) and thus *T_z_* is zero.

### 2.5 Model parameter estimation

As outlined above, model parameters are estimated by matching the simulated LV deformation with that observed in each of the diastolic MRI frame *i* during diastole (*i* ∈ [1, *n*] is the diastolic frame number, and *n* is the total number of diastolic MRI frames). The first diastolic frame (*i* = 1, i.e., the beginning-of-diastole frame) is defined as the minimum/zero pressure frame, while the last diastolic frame (*i* = *n*, i.e., the end-diastole frame) is defined as the frame synchronous to the R wave. The LV reference state (unloaded state, or stress-free state), defined as the state at which both the cavity pressure and myocardial AT are zero, is unknown. The state measured by the first diastolic frame is unlikely to be the reference state because while the pressure for this frame is assumed to be zero, the AT, particularly in the diseased cases, is likely to exist, and thus the the LV measured by the first diastolic frame is expected to be smaller than its reference state in terms of cavity volume.

**Fig. 5:**
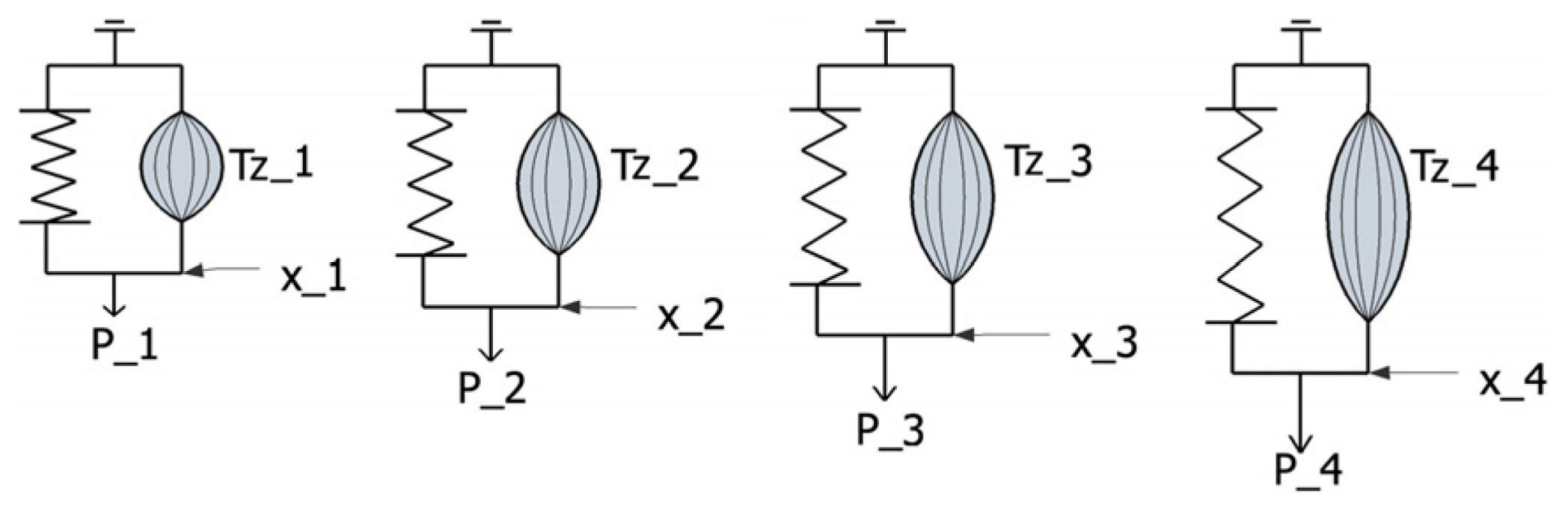
Schematic illustration of the inverse problem using the 1D model: 6 model parameters to be determined are active forces *T_z_* (1), *T_z_* (2), *T_z_* (3), *T_z_* (4) and two other variables illustrated in Fig. 3b – *K* (stiffness) and *x*_0_ (reference position), However, only four measurements (*x*_1_, *x*_2_, *x*_3_, *x*_4_) at four time points are available.

Thus the model parameters to be estimated includes constitutive parameters $\tilde{\text{C}},\;\text{ATs}\;\{{\tilde{T}}_{z}\left(i\right)\}i=1,\ldots ,n$ is the diastolic MRI frame number, as well as the reference state ${\tilde{\text{x}}}_{0}.$ These parameters are estimated by minimizing objective functions {*J_i_*} (defined below in Eq. (19)) based on the averaged geometrical difference between *i*th simulated mesh and the mesh fitted from the *i*th MRI frame. There are thus only *n* independent objective functions, from which *n* + 2 variables are to be determined. Therefore this inverse problem is under-determined. This concept is schematically illustrated in Fig. 5 using the previously introduced 1D system, where six model parameters are to be determined from only four measurements/equations at all time points.

#### 2.5.1 Further assumptions

To fully determine the system, two assumptions are added. Firstly, the AT is set to zero at the end-diastole, assuming the myocardium is fully relaxed

(17) $$T_{z}\left(n\right)=0.$$

Secondly, the unloaded state ***x***_0_ can be initially approximated (note this approximation will be later refined – see below) by the LV shape measured in one of the diastolic MRI frames (i.e., the reference MRI frame), where LV inflating pressure and contracting residual tension are assumed to be roughly balanced. *k* ∈ [1, *n* – 1] is defined as the reference frame number.

(18) $${\text{x}}_{0}∼{\text{y}}_{k}.$$

#### 2.5.2 Algorithmic description of parameter estimation procedure

Applying this approach, Algorithm 1 details the procedures for estimating constitutive parameters, reference state, and active tensions during diastole. This algorithm consists of five main steps as explained in the comments. Firstly, the assumption defined in Eq. (18) is applied to each diastolic MRI frame *k* before the ED frame, and the reference state ${\text{x}}_{0}^{k}$ is set to be the state measured by MRI frame *k* (line 3 of Algorithm 1). This reference state is then used in the second step to estimated the constitutive parameters ***C**^k^* (line 4), which are chosen as the optimal parameters with which the reference state can be best deformed to the ED state. These estimated constitutive parameters are in turn used in the third step to refine the reference state ${\text{x}}_{0}^{k}$, by deflating from the ED state (line 5). AT at each time point of MRI frames before the ED are estimated, using the pre-estimated reference state ${\text{x}}_{0}^{k}$ and constitutive parameters ***C**^k^* (line 7). Finally a criterion based on the physiological constraints of estimated AT, which is explained below, is devised to retrospectively choose the most sensible reference frame number (line 8) and the most plausible estimation of constitutive parameters, reference state, and active tensions (line 9).

The objective functions {*J_i_*} for estimating constitutive parameters and active tensions (used in lines 4 and 7 of Algorithm 1) are introduced below in Section 2.5.3. The details of methods for minimizing these objective functions {*J_i_*} are explained in Section 2.5.4. Finally the criterion in line 8 for choosing the most sensible reference frame number is described in Section 2.5.5.

**Algorithm 1.** Estimating constitutive parameters $\tilde{\text{C}},$ reference state ${\tilde{\text{x}}}_{0},$ and diastolic residual active tensions $\{{\tilde{T}}_{z}\left(i\right)\},$ given the MRI observations {***y**_i_*} during diastole and corresponding LV pressures {*P_i_*}. ${\tilde{\text{x}}}_{0}$ are the 3D coordinates of Gauss points at the reference state. ***y**_i_* are the coordinates of the same Gauss points given by the fitted meshes at time point *i* during diastole.

   **Data:** {***y**_i_*}, {*P_i_*}

   **Result:** $\tilde{\text{C}},$ ${\tilde{\text{x}}}_{0},$ $\left\{{\tilde{T}}_{z}\left(i\right)\right\}$

1 **begin**

          // Try each frame *k* before the ED as the reference frame

2        **for** *k*=1 to *n* − 1 **do**

                // Step 1: First set reference state ${\text{x}}_{0}^{k}$ to be the state measured by MRI frame *k*

3             ${\text{x}}_{0}^{k}={\text{y}}_{k}$

                // Step 2: Estimate constitutive parameters *C^k^* from ED measurement ***y**_n_*

4             ${\text{C}}^{k}=\underset{\text{C}}{\text{arg}\min}\;J_{n}\left({\text{x}}_{n},{\text{y}}_{n}\right),\text{where}\;{\text{x}}_{n}=𝕄\left(x_{0}^{k},\text{C},P_{n},T_{z}\left(n\right)=0,{\text{y}}_{n}^{B}\right)$

               // Step 3: Refine the estimation of refrence state ${\text{x}}_{0}^{k}$ by deflation

5             ${\text{x}}_{0}^{k}={𝕄}^{-1}\left({\text{y}}_{n},{\text{C}}^{k},P_{n},T_{z}\left(n\right)=0,{\text{y}}_{n}^{B}\right)$

6             // For each diastolic frame *i* before the ED

               **for** *i*=1 to *n* − 1 **do**

                     // Step 4: estimate AT $T_{z}^{k}\left(i\right)$ from ***y****_i_* using result from line 4 & 5

7                   $T_{z}^{k}\left(i\right)=\underset{T_{z}}{\text{argmin}\;}J_{i}\left({\text{x}}_{i},{\text{y}}_{i}\right),\;\text{where}\;{\text{x}}_{i}=𝕄\left({\text{x}}_{0}^{k},{\text{C}}^{k},P_{i},T_{z},{\text{y}}_{n}^{B}\right)$

          // Step 5: choose retrospectively which MRI frame should be initially assumed to be the refrence frame using AT-based criterion (eq. 20)

8       *k* = AT-Criterion$\left(\left\{T_{z}^{k}\left(i\right)\right\}\right)$

9       $\left[\tilde{\text{C}},{\tilde{\text{x}}}_{0},\left\{{\tilde{T}}_{z}\left(i\right)\right\}\right]=\left[{\text{C}}^{k},{\text{x}}_{0}^{k},\left\{T_{z}^{k}\left(i\right)\right\}\right]$

#### 2.5.3 Objective functions for estimating **C** and **T**_z_

The objective functions {*J_i_*} for estimating constitutive parameters and active tensions (used in lines 4 and 7 of Algorithm 1) are defined as the averaged distance between equivalent Gauss point *g* ∈ [1, *G*] of the simulated (***x**_ig_*) and fitted (***y**_ig_*) meshes at time point of *i*th MRI frame, i.e.

(19) $$J_{i}({\text{x}}_{i}.,{\text{y}}_{i}.)=\sqrt{\frac{1}{G}\sum_{g}\|{\text{x}}_{ig}{-{\text{y}}_{ig}\|}_{{𝕃}^{2}}^{2},}$$

where *i* ∈ [1, *n*] is the diastolic MRI frame number, g enumerates the index of Gauss points embedded inside each mesh volume (typically 4th order, *G* = 768 per element), and ***x**_ig_* and ***y**_ig_* denote the spatial coordinates of a Gauss point *g* at time point of *i*th MRI frame in the simulated and fitted mesh respectively. Gauss points are the sample points used in the standard Gauss–Legendre quadrature scheme for computing numerical integration (Hunter and Pullan, 2001).

#### 2.5.4 Minimization method for estimating **C** and **T**_z_

Having defined the objective function, the estimation of parameters described in Algorithm 1 is reduced to two minimization problems. We solve these minimization problems using the method of parameter sweeps, in which simulations are performed with varying parameter sets and the optimal parameter set is chosen. This kind of method is embarrassingly parallel, and it enables us to explore the landscape of the objective function, which in turn, helps to characterize the problems of parameter identifiability.

The method of minimization for constitutive parameters ***C*** (line 4 of Algorithm 1) is a two-step procedure. Using the reformulated Guccione law defined in Eqs. (9)–(13), *C*_1_ and *α* are first optimized by choosing the global minimum point across a grid that regularly samples 2D parameter space, followed by optimization of *r*_3_ and *r*_4_ using the same parameter sweeps method. This two-step process is iterated until the estimated parameters are converged. As we reported previously (Xi et al., 2011b), this optimization approach reveals the landscape of the objective function with respect to *C*_1_-*α*, in which the two parameters are strongly coupled. Because of the coupling between *C*_1_ and *α*, *C*_1_ needs to be fixed during the parameter estimation process to allow the remaining parameters to be uniquely determined. *C*_1_ was fixed at 1, based on average value of previous estimates of the Guccione law in literature (see Table 1). How this assumption *C*_1_ = 1 affects the results of this study is provided in the results section.

The method of minimization for AT *T_z_* (line 7 of Algorithm 1) is implemented as parameter sweeps in 1D parameter space, which regularly sample the AT parameter in a typical range of [−10, 30] kPa. To reduce the parameter samples, we first start with a coarsely even distributed parameter samples (typically with an interval of 1 kPa), from which we choose the optimal parameter and refine it locally using a smaller interval (typically 0.033 kPa).

#### 2.5.5 AT criterion for selecting reference frame

In line 8 Algorithm 1, a criterion based on physiological constraints is devised to select the most plausible frame as the reference frame. If frame *k* is the reference frame, then the estimated AT is expected to be monotonically decreasing during diastole and be positive (meaning that the AT is a contracting force). That is,

(20) $$T_{z}^{k}\left(i\right)>T_{z}^{k}\left(j\right),\;\text{for}\;\text{any}\;i>j;\;\text{and}\;T_{z}^{k}\left(n-1\right)>0.$$

Starting from *k* = 1, the first frame satisfying this criterion is chosen. We have tested and demonstrated the validity of this criterion using synthetic data where the ground-truth is known (details are provided in Appendix B.2).

## 3 Results

Our methodology is applied to three clinical cases. For each case, the processing time is approximately 40 min for the motion tracking, 1 min for the dynamic meshing, and 3 h for the parameter estimation. We used a highly optimized cubic-Hermite elements based mechanical simulation code (Land et al., 2011), running on a standard desktop computer (four 2.5 GHz cores and 4 GB RAM).

### 3.1 Dynamic meshing

**Fig. 6:**
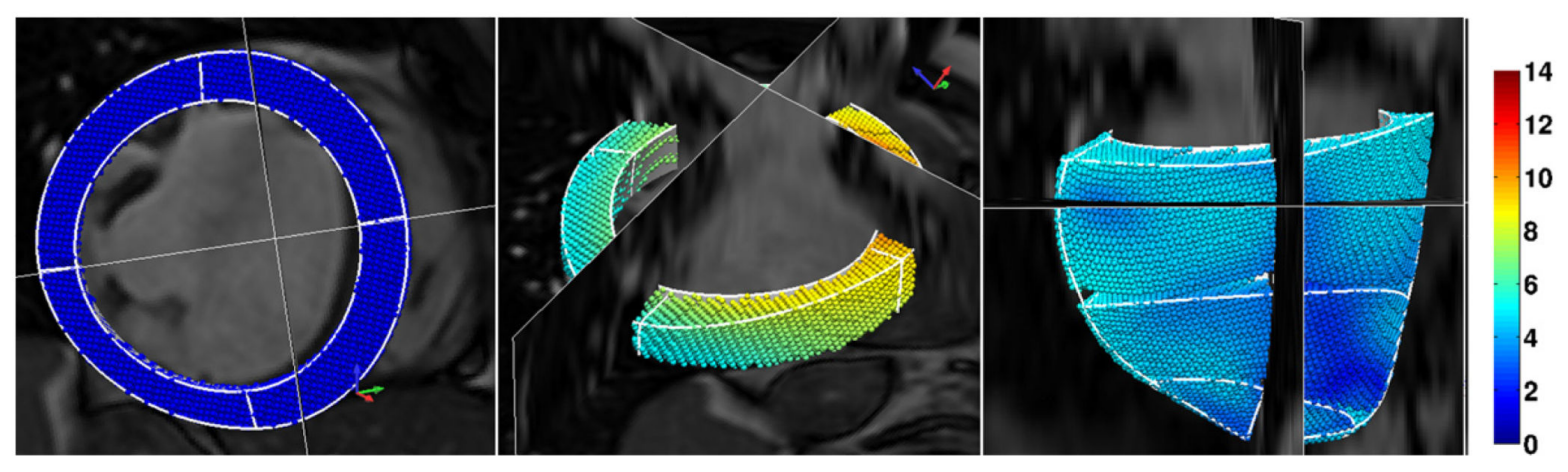
Results of dynamic meshing stage in Fig. 1 (the diseased case 1, visualized with the cine MRI from different views, corresponding to frames 1, 9 and 17 in a heart cycle starting with R wave). For visualization purpose, the mesh is shown together with the cine MRI. However, the meshes are reconstructed from the displacements of data points (embedded in the mesh) extracted from the combined tagged and cine MRI. The color represents the magnitude of displacement referencing to end-diastole in mm. (For interpretation of the references to color in this figure legend, the reader is referred to the web version of this article.)

Fig. 6 shows the fitted CH geometrical meshes for patient case 1, which are automatically constructed over a heart cycle following the methods outlined in Section 2.3 The residual of this fit (i.e., components of the fitting residual vector ***Z**_i_* – ***H***(Ξ)***U**_i_*) in Eq. (3) has a zero mean, standard deviation of 0.28–0.53 mm, and in general no obvious spatial correlations. As a result of this process, the displacements of discrete data points, which are extracted from the MRI data, are smoothed and regularized into the local material coordinates (model space). This representation of the deformation in model space enables the analysis of strain and stress to be performed in local material coordinates using standard finite element (FE) theory, provides patient-specific kinematic boundary conditions, and makes the comparison to FE model simulations straightforward.

### 3.2 Estimated constitutive parameters

Table 1 lists the estimated constitutive parameters $\left(\tilde{\text{C}}\right)$ for the three clinical cases. The results indicate consistently that the myocardium of two diseased patients is about threefold stiffer than the healthy case.

### 3.3 Estimated diastolic AT

**Fig. 7:**
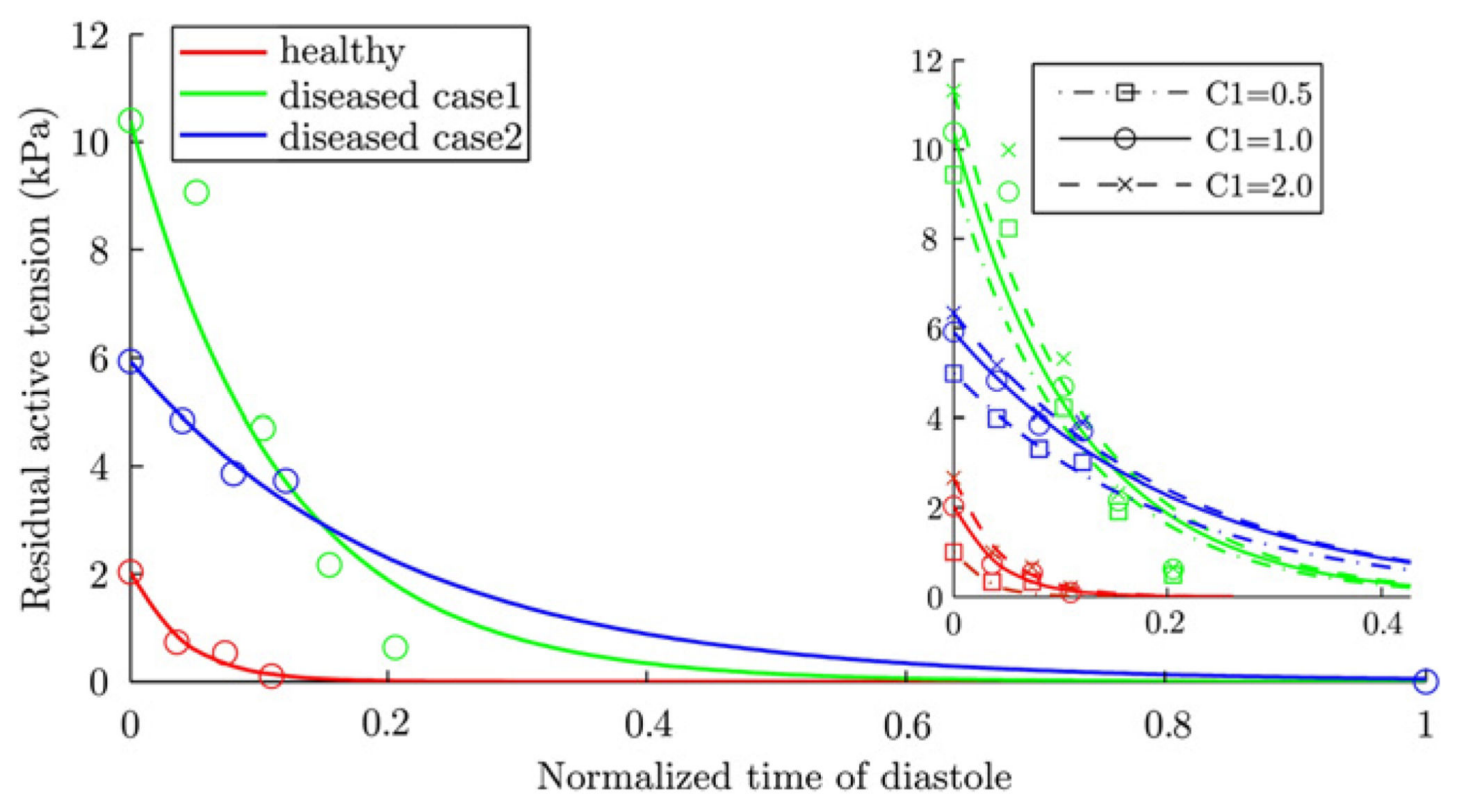
The estimated residual diastolic active tensions (*T*_z_ term defined in Eq. (16)) with its sensitivity against *C*_1_ assumption shown in the right panel. The data points show the optimized values of residual tension for each frame. The lines are the exponential fits to the data points. The time-line is the normalized time in a heart cycle, starting with end-diastole. Because limitations in clinical data acquisition protocol, tagged MRI only covers roughly one third of the early diastole. The residual AT of the two patient cases are significantly higher than the healthy one, indicating a delayed tension decay. The differences among the estimated AT using *C*_1_ = 0.5, 1.0, and 2.0 are very small, and this variability introduced by varying *C*_1_ is much smaller than the difference across patsient cases.

In Fig. 7, the color-coded points show the estimated residual AT $\left\{{\tilde{T}}_{z}\left(i\right)\right\}$ at all time points of diastole for the three cases. The relaxation (i.e., the tension decay) profiles, which in Fig. 7 are the exponentially fitted lines to the data points, of the patient cases show AT to be significantly different when compared to the healthy case.

To test the sensitivity of the residual AT profile against the assumption of *C*_1_, we also estimated the tension profiles for each case using *C*_1_ = 0.5 and *C*_1_ = 2.0, based on the variability of *C*_1_ in the literature. The right panel of Fig. 7 shows these results. In each of the three cases, the variability introduced by varying *C*_1_ is much smaller than the difference across patient cases. In addition, the estimated AT increases with the increase of *C*_1_, which is a result of the decreased non-linearity in the Guccione constitutive law due to the compensatory decrease of *α*.

### 3.4 Model simulation with estimated parameters

**Fig. 8:**
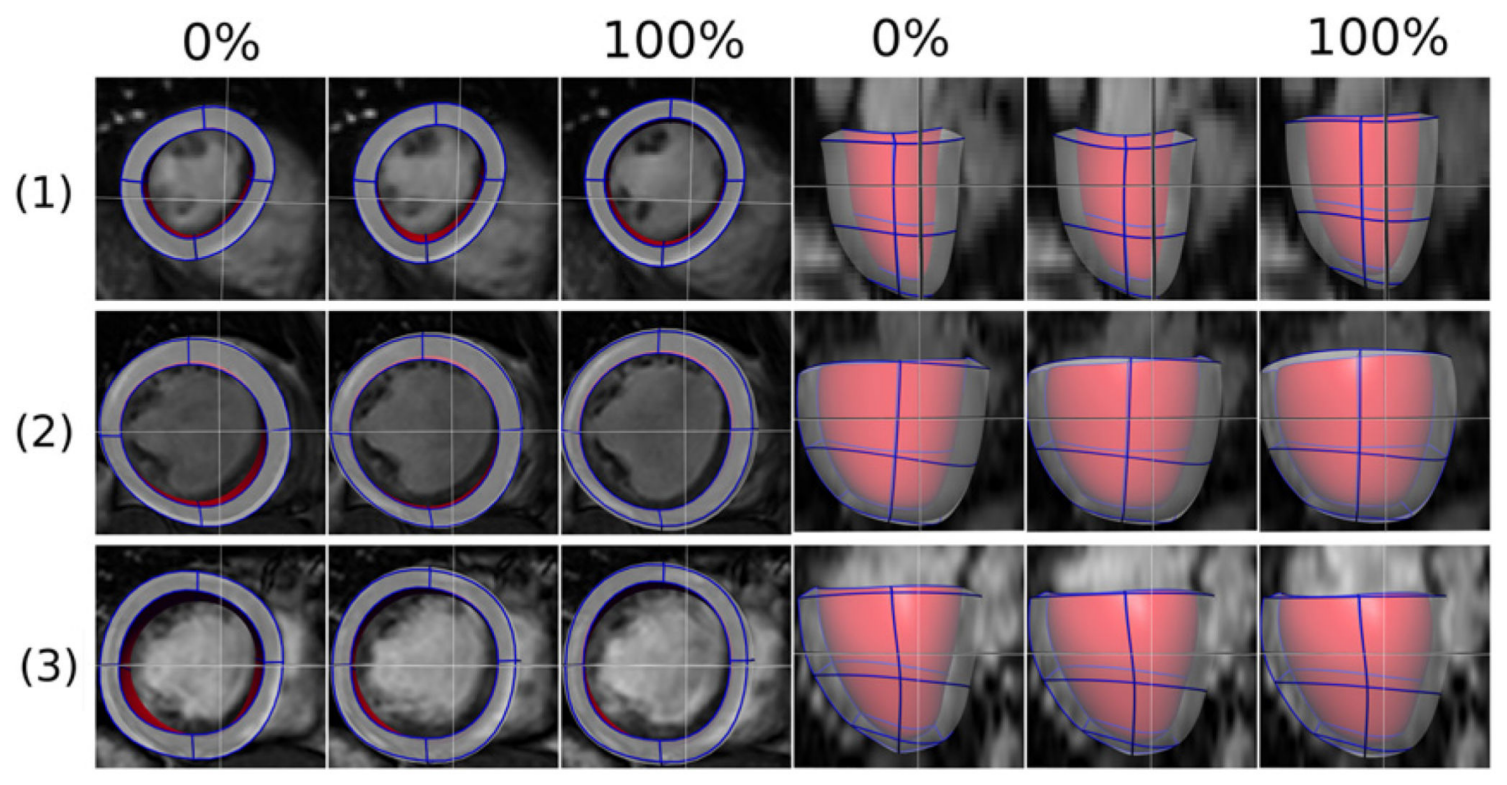
The final simulated meshes for the diastole process of the three cases (1 is healthy, and 2, 3 are diseased 1 and 2), using estimated constitutive parameters, reference (unloaded) state, and AT parameters. These simulated meshes are visualized with the corresponding cine MRI frames. The meshes are shown in short axis view (left three columns) and long axis view (right three columns), each view consisting of the simulation results at 0%, 15%/24%/16% and 100% of the diastole phase.

Fig. 8 shows the final model simulation results for the three clinical cases, using the estimated constitutive parameters, reference state and active parameters. As reported previously in Table 1, the residual at the ED (100%) is 1.78, 1.58 and 1.39 mm for the healthy case, disease 1 and 2 respectively.

## 4 Discussion

In this study, we present a method for estimating diastolic active and passive myocardium parameters – namely the myocardial constitutive properties and diastolic residual AT – from combined cine and tagged MR images and cavity pressure measurements. Applying this method to three clinical cases, the diastolic AT estimation results show a significant difference between healthy and two diseased cases, which may provide an interesting starting point for further clinical research. Below we discuss the issues related to the estimation of myocardial constitutive parameters, and the sensitivity of key steps in our methods on AT estimation results.

### 4.1 Issues related to the estimation of constitutive parameters

#### 4.1.1 Parameter identifiability and C_1_ assumption

Clinically, tagged MRI data covering the whole of diastole is difficult to record, while the end-diastolic frame (typically the first frame when synchronized with the R-wave of ECG) is always available. Thus, it is desirable to characterize myocardial stiffness using displacements (relative to the reference state) extracted from the end-diastolic frame of tagged MRI. However, the issues associated with the identification of the Guccione parameters using displacements extracted from only one MRI frame has been already reported by several previous studies (Omens et al., 1993; Augenstein et al., 2005, 2006; Xi et al., 2011b). For this reason, Omens et al. (1993) only estimated *C*_1_ and the ratio of *C*_2_ : *C*_3_. Augenstein et al. (2006) has reported identifiability problems in the form of a correlation matrix, which interestingly showed a low level of linear correlation between *C*_1_ and *α*. Our previous study (Xi et al., 2011b) further explicitly reveals the non-linear (or log-linear) correlation between *C*_1_ and α $C_{1}^{a}\alpha =b$, where *a* and *b* are constant).

In order to obtain the complete set of unique parameter values, multiple tagged MRI frames during diastole can be used. Each frame can provide information from which a distinct curve of the form $C_{1}^{a}\alpha =b$ can be estimated. For example, the synthetic results provided in the appendix (Fig. B.13a) show that all *C*_1_-*α* curves estimated from different MRI frames intersect at the ground-truth parameter point. Augenstein et al. (2005) has also showed that five MRI frames were sufficient to characterize the material parameters to within 5% error. However, results with patient data showed that there is not a unique intersection point in the parameter space, and the presence of residual AT is likely the main reason. This is illustrated by Fig. B.13f where the rapid decrease of residual AT between early diastolic frames leads to estimation of softer myocardial stiffness in fiber direction. Thus, the high level of residual AT compromises the feasibility of estimating full set of Guccione parameter using early-diastole frames, particularly in the clinical cases where diastolic pathologies are indicated.

For this reason, it is assumed that *C*_1_ is one, based on the average value reported in literature (see Table 1). For the range of the variability in *C*_1_ as reported in literature, our analysis was consistent in its ability to identify a significant difference between tension profiles of the healthy and diseased cases as shown in Fig. 7.

#### 4.1.2 Choice of constitutive laws

There are a number of existing constitutive models for passive cardiac mechanics (e.g., see Holzapfel and Ogden, 2009 for a comprehensive survey). In particular, to solve the parameter identifiability problem, a number of researchers have proposed new constitutive laws, such as the 5-parameter polynomial form strain energy function by Humphrey et al. (1990), and the optimized strain energy function based on uncoupled strain attributes by Criscione et al. (2001). In our study, in order to facilitate the comparison of estimated parameter values with those reported in literature, we employed and reformulated the Fung-type Guccione’s law, which is consistent with the widely-used approach in the cardiac modeling community for both forward simulation of cardiac mechanics and inverse model parameter estimation (Omens et al., 1993; Augenstein et al., 2006; Wang et al., 2009; Sun et al., 2009; Niederer and Smith, 2009; Xi et al., 2011a). Nevertheless, the general principles of our proposed method for estimating diastolic AT apply to other constitutive laws as well.

### 4.2 Sensitivity of individual components in our methods on AT

The Algorithm 1 outlined in Section 2.5.2 is the core of our AT estimation method. In this method, we proposed to estimate not only the passive constitutive parameters (step 2 in Algorithm 1), but also the reference state by deflating from the ED state (step 3 in Algorithm 1). Both of these two steps depend on assumption defined in Eq. (18) – the state measured by *k*th diastolic MRI frame is close to the reference state (i.e., the step 1 in Algorithm 1). The validity of our proposed approach clearly relies on both Assumption 18 and individual step of Algorithm 1, which in turn motivates the analysis of the contribution to the accuracy of the final AT result.

**Fig. 9:**
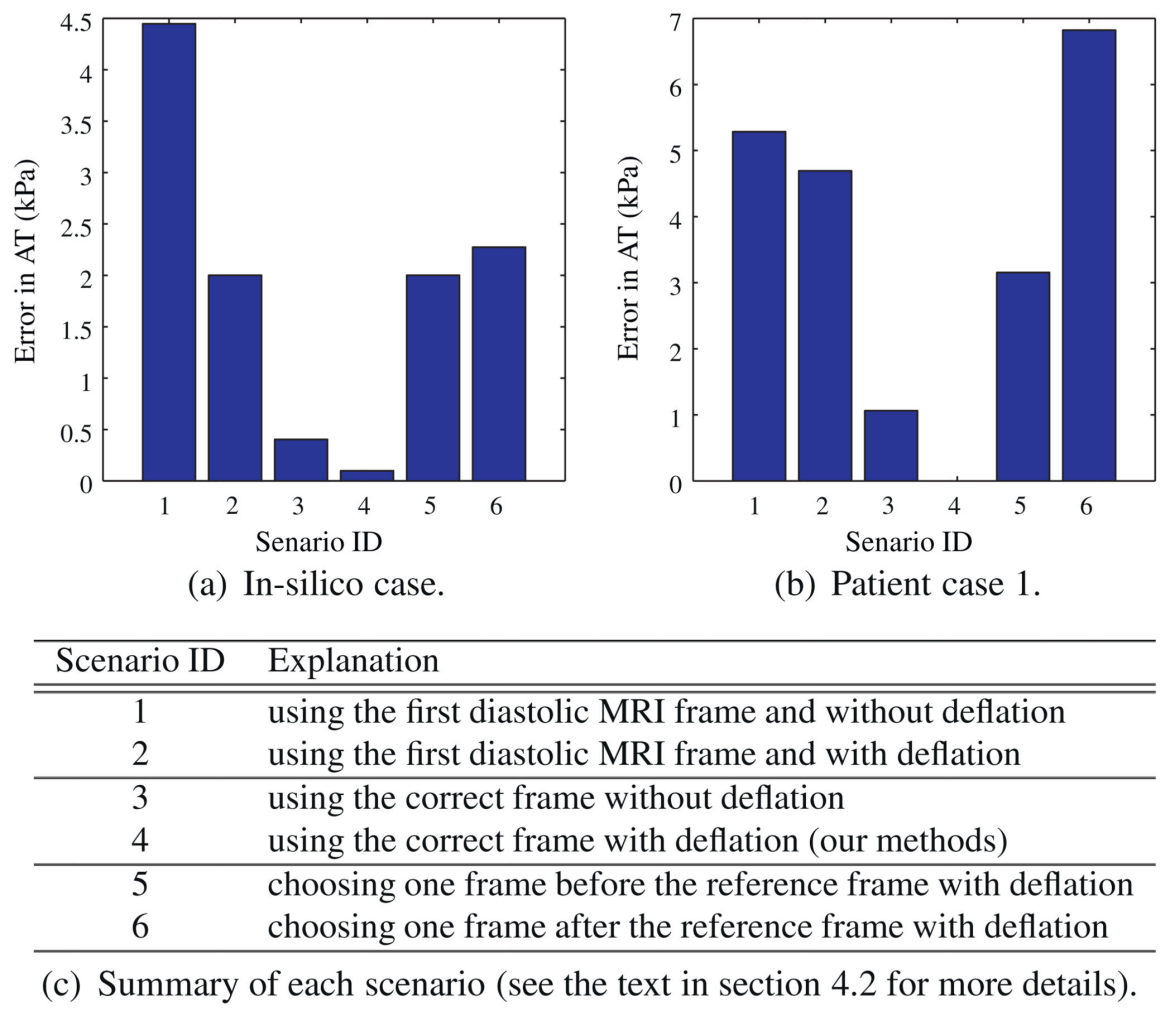
The error of AT introduced under six scenarios for an in silico case and the patient case 1, to assess the importance of choice of reference frame and deflation step in our methods (see the text in Section 4.2 for details). The error of AT (in kPa) is defined as the root of mean squared error (RMSE) between AT estimated in each scenario and the known ground-truth (in silico case)/AT estimated in scenario 4 (patient case 1). In the in silico case, the measurement used are the simulated meshes, which are produced by our model using a linearly increasing LV pressure (0.33, 0.67, 1.00, 1.33, 1.67 and 2.00 kPa) and exponentially decaying AT (8.00, 2.35, 0.68, 0.21, 0.05, and 0 kPa).

In Fig. 9, we investigate, using six scenarios, the analysis of the relative importance of

(1) step 1 – the choice of reference frame number *k* and
(2) step 3 – the deflation step of estimating reference state.

Fig. 9a and b shows the errors for in silico case and patient case 1 respectively in each of six scenarios. Scenario 1 is the simplest form of Algorithm 1, in which *k* = 1 and the deflation step is omitted. This scenario is effectively equivalent to directly using the state measured by the first diastolic MRI frame as the unloaded reference state, a situation that is only true if the residual AT and LV cavity pressure are both zero. In scenario 2, the deflation step is included on top of scenario 1. Similarly, the frame number *k* determined by the criterion of Eq. (20) is used in scenarios 3 (without deflation) and 4 (with deflation). Note that scenario 4, which uses the whole Algorithm 1, produces the gold standard result for comparison for the patient case where ground-truth AT is unknown. Scenarios 5 and 6 (both with deflation) use one frame before and after the correct reference frame used in scenarios 3 and 4.

These results indicate that the inclusion of deflation step improves the accuracy of AT estimation. Nevertheless, the deflation is relatively less important when choosing the correct reference frame since the state at correct reference frame is already very close to the reference state. In addition, the choice of *k* is the most important step, because this affects both the estimation of constitutive parameters and reference state. In particular, if *k* = 1 and the deflation step is omitted (scenario 1), the influence of AT is not considered (Wang et al., 2009; Xi et al., 2011b), possibly leading to biased estimation of constitutive parameters.

### 4.3 Limitations and future work

#### 4.3.1 Limitations

While our results appear promising, it is important to note that there are a number of limitations in our approach. The rule-based fiber distribution of our LV model does not incorporate directly the patient-specific measurements, and this may influence the estimation of material anisotropies and the accuracy of AT estimated. To address this issue, we are currently in the process of building a human fiber model by acquiring and post-processing in vivo diffusion-tensor images. In addition, because we have not included the mechanical effects of organs around left ventricle, kinematic displacements are imposed as boundary conditions at both the LV apex and the base in order to constrain the predicated movement. However, this is likely to affect the finite elasticity solution and motion prediction in the free wall, possibly leading to a biased material property estimate. Ideally models of pericardium, right ventricle, and atrium would be included. However, such additions would clearly be at the cost of increasing complexity in both model simulations and inverse parameter estimation.

Limitations in the measurements include the pressure data recordings which have a level of uncertainty due to the calibration error, in part because only the $\frac{dP}{dt}$ trace was available without a reference to the absolute value of *P*. To account for this gap in the data, we assumed that the minimum pressure (at the beginning of diastole) is zero, based on the data reported by Wang et al. (2009). Another limitation regarding pressure data is that the clinical protocol limits the pressure measurements to being recorded separately from the MR imaging. While the patients were in the same physical position in both MRI scan and catherization procedure (rest on their back), it is possible there might be small changes in haemodynics states in the pressure and imaging recordings due to the insertion of the pressure catheter lead and the surgical anesthetization.

#### 4.3.2 Future work

We choose a robust but computational expensive approach to sample the parameter space. As explained above, this enabled us to fully explore and understand the coupling relationships between parameter values. In the future, it would be possible to adopt more sophisticated but computationally effective approaches to directly estimate the coupling coefficients *a* and *b*, such as SQP (Augenstein et al., 2005) or filtering approaches (Xi et al., 2011a).

Finally, this study brings about a significant requirement on the completeness and accuracy of various clinical data. Limitations of the patient data used in this study restrict the analysis to early filling, in which passive diastolic recoil is combined with the relaxation of AT. Since the tagged MRI measurements do not cover the period of pure passive filling, passive material properties are confounded with active relaxation. In the future, we plan to acquire additional clinical data sets with optimized protocols (e.g., whole-heart-cycle tagged MRI coverage, diffusion-tensor imaging for the patient-specific fiber distribution), in order to further invetigate and correlate our new indices with clinical diagnosis.

## 5 Conclusion

Our methods of integrating the clinical MRI and LV cavity pressure data across multiple measurement points in the diastole enabled us to provide, to our knowledge, the first attempt to estimate the diastolic residual active tension profile in human subjects, which has significant potential to provide an important metric characterizing diastolic heart failure. The results from our preliminary application of this method indicates that early diastolic residual AT in the two diseased cases are significantly higher than the normal cases, which may well indicate that myocardial relaxation (i.e., lusitropy) is impaired in those two patient cases.

## Supplementary Material

Appendices

## Figures and Tables

**Table 1:** Estimated constitutive parameters $\left(\tilde{C}\right)$ for one healthy case and two patient cases, and comparison to studies in literature.

–	ESV (ml)	EF (%)	*C*_1_	*C*_2_	*C*_3_	*C*_4_	Residual^a^
Healthy case	67	51	1.0	19.13	10.67	12.76	1.78
Case 1	345	16	1.0	53.44	22.01	29.34	1.58
Case 2	186	17	1.0	50.50	16.83	27.19	1.39
Augenstein et al. (2005), dog	–	–	1.5^b^	11.1	1.76	10.0	–
Wang et al. (2009), dog	–	–	0.831	14.3	4.49	0.762	1.81
Omens et al. (1993), dog	–	–	1.2	26.7	2.0	14.7	–
Okamoto et al. (2000), dog	–	–	0.51	67.07	24.16	21.60	–
Omens et al. (1993), rat	–	–	1.1	9.2	2.0	3.7	–
Walker et al. (2005), sheep	84.7	21.6	0.233	49.25	19.25	17.44	–

EDP, end-diastolic LV cavity pressure; ESV: end-systolic LV cavity volume; EF, ejection fraction.

a The root-mean-squared-error (RMSE) in mm between simulated and fitted mesh at end-diastole over free wall.

b *C*_1_ = 3.0 in this study is defined with a multiplier of $\frac{1}{2}$.

## Footnotes

1 IRTK, http://www.doc.ic.ac.uk/~dr/software/.
