# Supplementary material for: The estimation of patient-specific cardiac diastolic functions from clinical measurements
Source: Med Image Anal. Author manuscript; Available in PMC 2019 Sep 30. (PMC6768802; doi:10.1016/j.media.2012.08.001)
Supplement: Appendices [file EMS84391-supplement-Appendices.pdf]

Appendix A. Constitutive parameter coupling

A.1. Derivations of the C_1-α coupling

The Guccione parameters are identified by matching the simulated deformed mesh(s) to the fitted mesh(s) from the MRI frame(s). However, from the current clinical data, the Guccione parameters cannot be reliably identified using only the ED frame in the sense that an increase of C_1 can be compensated for by a decrease of C_2–C_4 or α.

To explicit reveal this problem, our goal is to find the coupling direction $\boldsymbol{d}$ in C_1-α space (if any) along which two sets of different parameters would render the same (or very similar) mechanical simulation. In the following analysis, the hydrostatic tensor term can be safely eliminated. From the numerical solution perspective, given the same external loading conditions (external forces) and temporary trial solution of a strain tensor (displacement DOF), the whole stress tensor (deviatoric plus hydrostatic) should be always the same as long as the deviatoric stress tensors are the same. This is because the solver for the hydrostatic term is only concerned with the strain and residual stress, not the material parameters.

For notations, $\mathbf{T}$ is the deviatoric second Piola–Kirchhoff stress tensors. $\mathbf{E}$ is the Green–Lagrangian strain tensor.

$$\mathbf{T} = \frac{\partial W}{\partial \mathbf{E}} = 2C_1 e^Q \begin{pmatrix} C_2 & C_4 & C_4 \\ C_4 & C_3 & C_3 \\ C_4 & C_3 & C_3 \end{pmatrix} \circ \mathbf{E}, \tag{A.1}$$

where $\circ$ is the operator for element-wise product (or Hadamard product).

The material elastic tensor $\mathbf{K}$ for Guccione's constitutive law is defined as:

$$\mathbf{K} = 2C_1 e^Q \begin{pmatrix} C_2 & C_4 & C_4 \\ C_4 & C_3 & C_3 \\ C_4 & C_3 & C_3 \end{pmatrix} = 2C_1 \alpha e^Q \begin{pmatrix} r_2 & r_4 & r_4 \\ r_4 & r_3 & r_3 \\ r_4 & r_3 & r_3 \end{pmatrix}. \tag{A.2}$$

The coupling direction $\boldsymbol{d}$ in C_1-α space (if it exits) is defined where the directional gradient of $\mathbf{K}$ along d is a zero-tensor. Intuitively the material response (stress–strain relationship) is the same along this (local) direction.

$$\begin{pmatrix} \frac{\partial \mathbf{K}_i}{\partial C_1} \\ \frac{\partial \mathbf{K}_i}{\partial \alpha} \end{pmatrix} \cdot \boldsymbol{d} = 0, \quad i = 1, \ldots, 9. \tag{A.3}$$

These nine conditions from the above equations are combined to be one constraint independent of r_2–r_4:

$$\begin{pmatrix} \alpha e^Q \\ C_1 e^Q + C_1 \alpha \frac{\partial e^Q}{\partial \alpha} \end{pmatrix} \cdot \boldsymbol{d} = e^Q \begin{pmatrix} \alpha \\ C_1 (1 + \alpha \frac{\partial Q}{\partial \alpha}) \end{pmatrix} \cdot \boldsymbol{d} = 0. \tag{A.4}$$

A.1.1. The existence of the coupling direction

The coupling direction $\boldsymbol{d}$ exists if and only if Eq. (A.4) has a solution. The term $\frac{\partial Q}{\partial \alpha}$ in Eq. (A.4) can be further expanded by using Eqs. (8) and (13), i.e.

$$\frac{\partial Q}{\partial \alpha} = \frac{\partial \left(\sum_{i,j} \alpha r_{ij} E_{ij}^2 \right)}{\partial \alpha} \tag{A.5}$$

$$= \frac{Q}{\alpha} + \sum_{i,j=1} \alpha \left(2 r_{ij} E_{ij} \frac{\partial E_{ij}}{\partial \alpha} \right), \quad i,j = 1,2,3, \tag{A.6}$$

where r_{ij} denotes the corresponding elements of rightmost matrix in Eq. (A.2), and E_{ij} are the Green–Lagrange strains in fiber ($f := 1$), sheet ($s := 2$) and sheet normal ($n := 3$) directions. The second term $\frac{\partial E_{ij}}{\partial \alpha}$ is varying at different (C_1, α) points, and thus dependent on the coupling direction $\boldsymbol{d}$ which we are solving for. Therefore Eq. (A.4) is non-linear.

The solution of a non-linear equation does not necessarily exist. Thus the "zero-coupling direction" (the direction along which the deformation is exactly the same) may not exist. However, as evidenced by Fig. A.10 and reported by Xi et al. (2011a), there does exist a "principle-coupling direction" – the direction along which the change is very close to zero and significantly smaller than other directions.

A.1.2. The approximated exponential coupling curve

If we ignore the second non-linear term in $\frac{\partial Q}{\partial \alpha}$, that is,

$$\frac{\partial Q}{\partial \alpha} \approx \frac{Q}{\alpha},$$

Eq. (A.4) can be simplified as

$$e^Q \begin{pmatrix} \alpha \\ C_1 (1 + Q) \end{pmatrix} \cdot \boldsymbol{d} = 0. \tag{A.7}$$

Therefore the coupling curve in (C_1, α) space roughly has the tangent direction $\boldsymbol{d} = (\frac{C_1}{\alpha} - \frac{1}{1+Q})^T$, which indicates the curve is

$$C_1^{\frac{1}{1+Q}} \alpha = b, \tag{A.8}$$

where b is a constant.

Note that we ignored the second non-linear term in Eq. (A.6) and use the assumption that the slope of the curve $\frac{1}{1+Q}$ is constant when we derive the approximated yet simple curve expression (Eq. (A.8)). While these are mathematical approximations, in practice it already provides a good agreement with the coupling curves fitted numerically (see Fig. A.10).

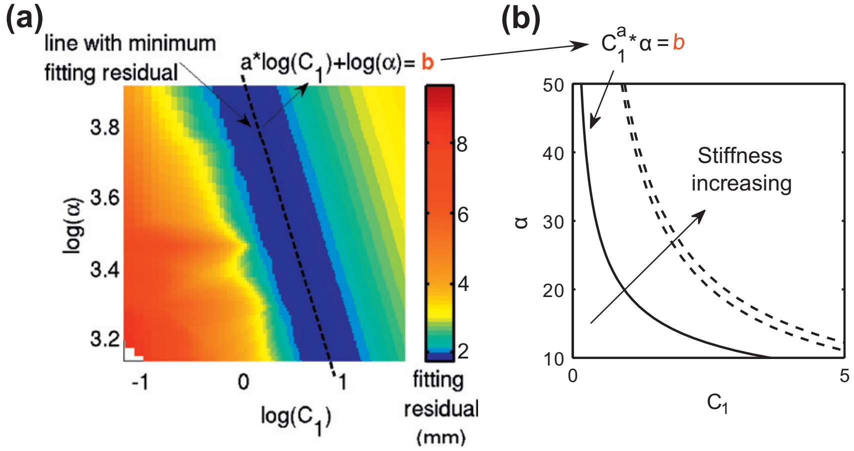

Fig. A.10. Parameter optimization and C_1-α curve fitting. (a) The landscape of objective function using only the ED measurement (i.e., the Eq. (19) when $i = n$) with respect to C_1-α for patient case 1. This landscape is obtained from our two-step optimization process. The color represents the magnitude of the objective function in mm. The dark blue valley indicates a straight line with equal optimal parameter fits. (b) A linear line in the log-scale space with the form of $a * \log(C_1) + \log(\alpha) = b$ is fitted to optimal fitting valley in (a). This line in the log-space corresponds to the curve of $C_1^a\alpha = b$ in (b). (For interpretation of the references to color in this figure legend, the reader is referred to the web version of this article.)

A.2. The landscape of minimization objective function

The landscape of objective function using only the ED measurement (i.e., the Eq. (19) when $i = n$) with respect C_1-α and r_3—r_4 are shown in Fig. A.10 and Fig. A.11 respectively. These landscape empirically demonstrated that, from the parameter estimation point of view, C_1 and α are coupled while r_3 and r_4 are relatively uncoupled.

A.3. The theoretical implication of incorporating multiple measurements

C_1 and α are coupled which sufficiently render the optimization problem effectively ill posed. However, the coupling relationships at different deformation state are different, because $\frac{1}{1+Q}$ is dependent on the strains. Thus we can improve our estimation by comparing the simulated deformations with *multiple* displacement measurements instead of only the end-diastolic one.

The theoretical implication of incorporating incorporating multiple measurements is intuitive. The Guccione constitutive law is a Fung-type or exponential-type strain energy function. Its linear (C_1) and exponential (α) coefficients are coupled at one measurement point, but differently coupled across multiple measurements. Fig. A.12 shows changes of the Guccione strain energy with respect to C_1-α, where the increase of measurement points improves the identifiability of the parameters.

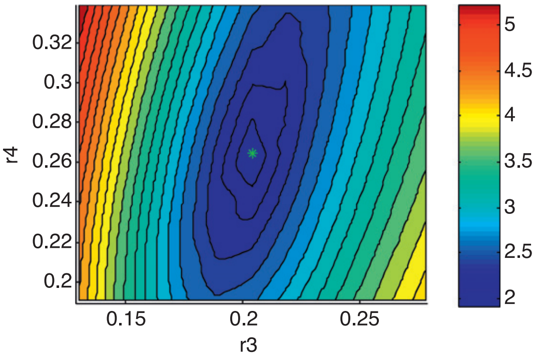

Fig. A.11. Objective function plot with respect to r_3 and r_4 for patient case 1. The color represents the magnitude of objective function in mm. The correlation between r_3 and r_4 is 0.53. Unlike the strong C_1-α coupling, the optimization problem in r_3–r_4 space is better posed.

Appendix B. Estimation of diastolic active tension

B.1. Motivation of accounting for diastolic AT in the model

Fig. B.13 shows the constitutive parameter estimation results without accounting for AT in the model, which, in turn, motivates the necessity of adding an AT component into the model to explain the deformation fields. These results are the estimation of four reformulated Guccione parameters – C_1, α, $r3$ and $r4$ ($r_2 = 1 - r_3 - r_4$), using observations of meshes fitted to different MRI frames or synthetically simulated meshes. Since C_1-α is coupled and cannot be uniquely identified from one displacement measurement, Fig. B.13a, b, d and f show C_1-α (coupling) curves, instead of only one unique (C_1, α) point. These exponential C_1-α lines are fitted separately, in log space, to a set of equally optimal parameters points (refer Fig. A.10 and Xi et al., 2011b for more details of the C_1-α curve and its fitting).

The in silico results (Fig. B.13a–c) are the estimation results from synthetically simulated meshes. In Fig. B.13a, varying LV endocardium pressure (0–2 kPa) are applied as loading conditions with zero AT within simulation, and C_1-α relationship is estimated separately from each of these measurements. In Fig. B.13b and c, the synthetic meshes are simulated with the same pressure (2 kPa) and varying AT (0–8 kPa), a C_1-α relationship is estimated separately from each of these measurements (Fig. B.13b), together with corresponding r_3–r_4 estimation results (Fig. B.13c).

The results for the healthy and diseased cases (Fig. B.13d–g) are obtained from the meshes fitted to different MRI frames. In this estimation process, the reference mesh is set to be the beginning-of-diastole frame (defined in Section 2.5 as the 1st diastolic frame whose LV pressure is assumed to be zero). This is because when AT is not considered in the model, the LV is unloaded if and only if LV cavity pressure is zero.

The result for the in silico case indicates that the inclusion of AT in the measurement would shift the C_1-α curve in parallel, as well as changing the r_2 (the stiffness ratio in fiber direction) consistently. This behavior is hardly noticeable in the healthy case, but is clearly demonstrated in the disease cases, which motivates the needs of considering AT for estimating parameters in the disease cases.

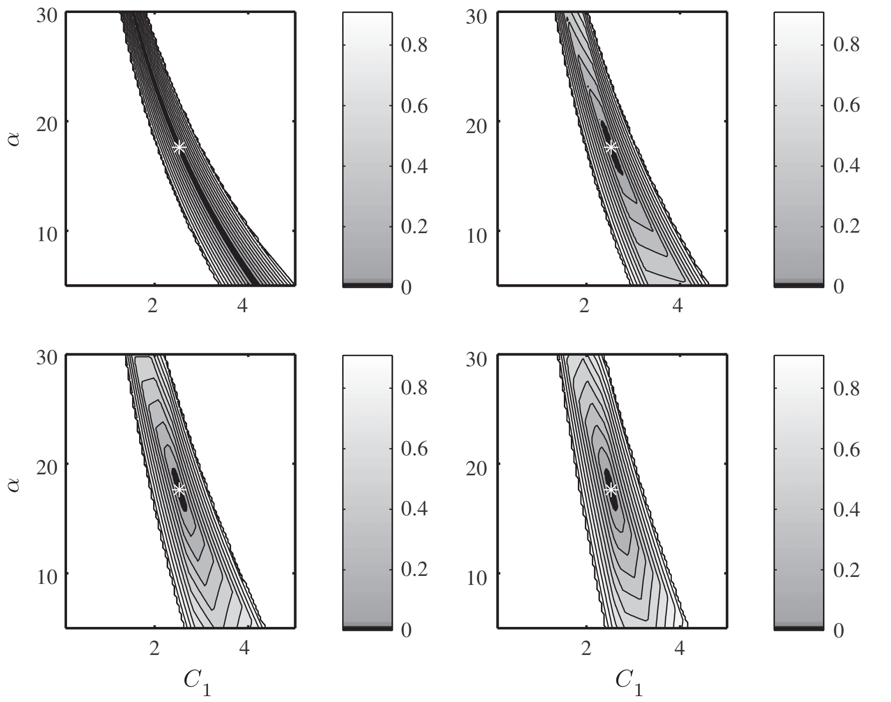

Fig. A.12. Changes of the Guccione strain energy with respect to C_1-α, averaged over 1, 2, 3 and 4 "measurement point(s)". The optimization problem becomes less ill defined with the increase of measurement constraints. This reveals the possibility of obtaining a unique global minimum solution of parameter estimation when incorporating multiple measurements.

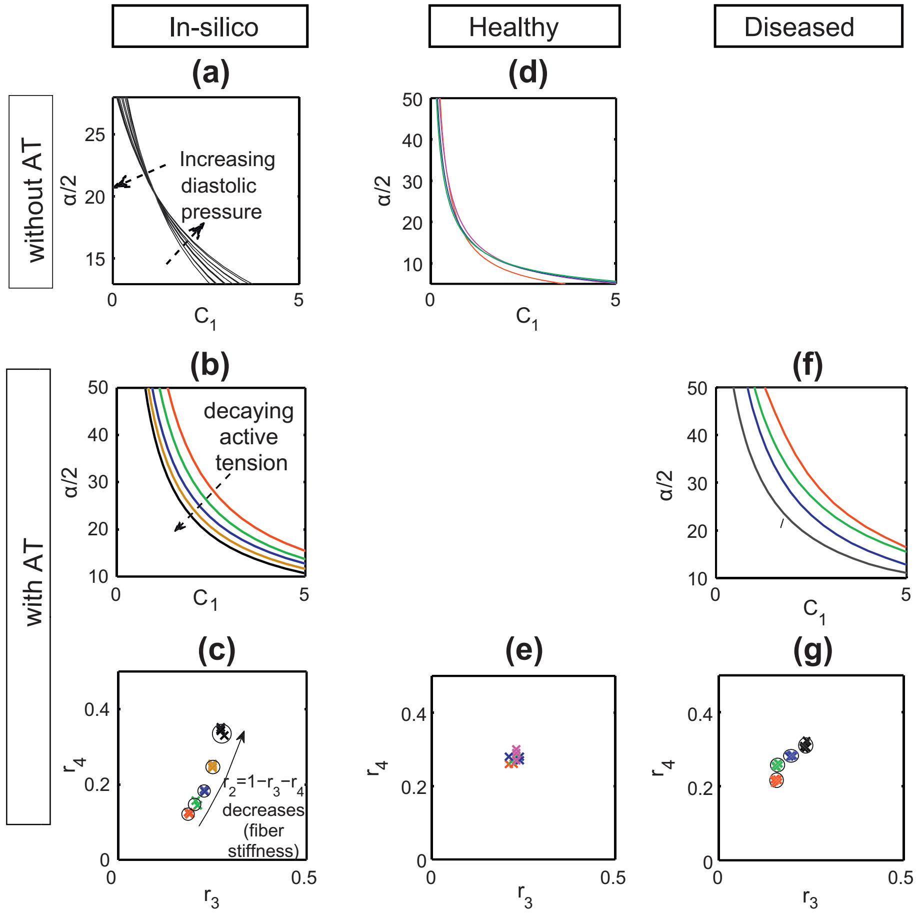

Fig. B.13. Constitutive parameter estimation without accounting for AT in mechanical model, showing that the *C1*-αcurves (subplots a, d, b and f) and $r_2 = 1 - r_3 - r_4$ (subplots c and g) estimated from different MRI frames are not constant. All the models assume zero AT as part of the parameter estimation process. The "with AT" plots refers to AT added to the in silico simulation (figure b) or assumed in the patient data (figure f), and please refer to Section B.1 for details.

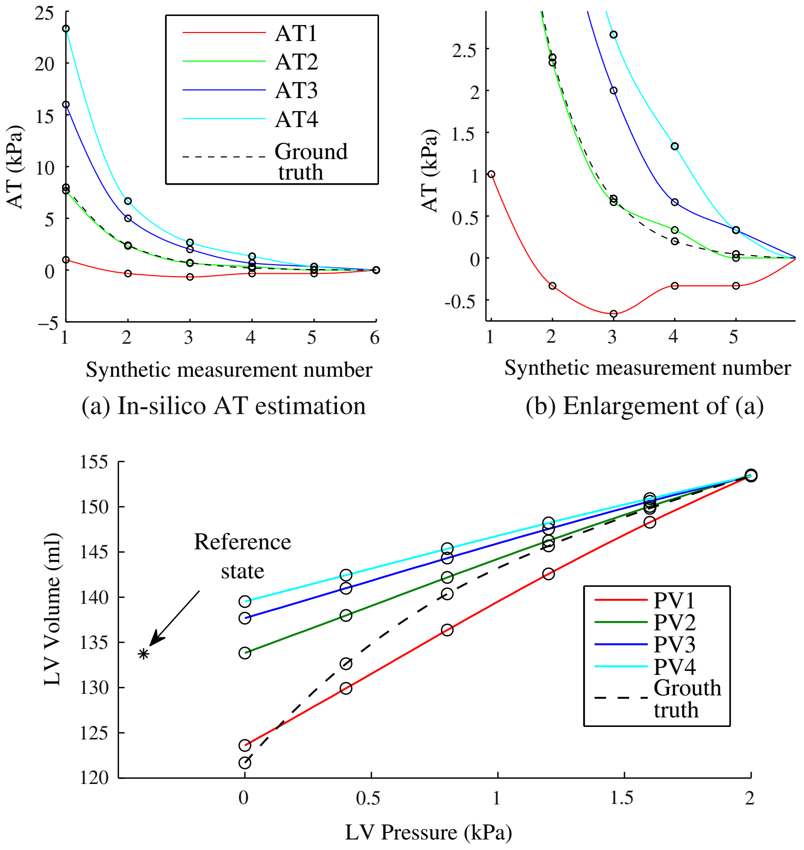

(a) In-silico AT estimation

(b) Enlargement of (a)

(c) PV curves (PV1-4) of the in-silico purely LV inflation simulations without AT.

Fig. B.14. In-silico AT estimation using different frames as the reference frame (a and b) and the corresponding simulated PV curves using estimated parameters without accounting for AT (c). In subplots (a) and (b), AT 1–4 are the in silico AT estimation results using the 1st, 2nd, 3rd and 4th measurement as the reference frame. In subplot (c), the corresponding PV 1–4 are produced by the pure passive inflation with estimated constitutive parameters and reference state. The difference between each of PV 1–4 curves and the ground-truth PV curve in subplot (c) corresponds to the sign of AT 1–4 in subplot (a). That is, AT are estimated to effectively match the ground-truth PV curve of the in silico measurements. The positive AT decreases the volume of LV while the negative AT increases the volume of LV (e.g., see AT 1 and PV 1). Note that the volume curves in subplot (c) are only for schematically illustrating the meaning of the sign of AT, and the actual minimized objective function for estimating the parameters is based on the 3D displacement of the LV (Eq. (19)), which is similar but not identical to the objective function based on LV volume.

B.2. Illustration of the criterion of selecting reference frame

Since each early diastolic MRI frame is initially assumed to be the reference frame in Algorithm 1, we use the physiological constraints on AT to devise a criterion (defined in Eq. (20)) to retrospectively choose which MRI frame should be the most correct reference frame. This criterion implies that AT is monotonically decreasing during diastole and non-negative (since a positive AT denotes a contracting force).

To demonstrate this idea, the same in silico case as previously described in Fig. 9 is used. In this in silico case, the measurement used are the six simulated LV meshes, which are produced by the mechanical model using a linearly increasing LV pressure (0.33, 0.67, 1.00, 1.33, 1.67 and 2.00 kPa) and exponentially decaying AT (8.00, 2.35, 0.68, 0.21, 0.05, and 0 kPa). The volume of these simulated meshes (the ground-truth PV curve) is shown in Fig. 9c. The AT estimation method (Algorithm 1) is applied and the estimated AT is shown in Fig. 9a and b, in which the AT 1–4 corresponds to the AT estimated using 1st, 2nd, 3rd and 4th frame as the reference frame (i.e., when $k = 1, 2, 3, 4$ in Algorithm 1).

According to the AT criterion defined in Eq. (20), frame 2 is selected as the reference frame and AT 2 of Fig. 9b is selected as the AT estimation result, which in turn, produces a very small error between estimated and ground-truth AT. Note that AT 3 and AT 4 also satisfy the criterion. However, as stated previously in the text explaining the AT criterion, only the first frame satisfying the criterion should be selected. The reason for this is demonstrated by Fig. 9a where the simulated PV curves using the mostly plausible reference frame (i.e., PV 2) should the one tangent to the ground-truth PV curve at the ED point. Subsequent PV curves (PV 3 and 4 using frame 3 and 4 as reference frame) would overestimate the stiffness in constitutive parameters (i.e., the slope of PV curve) while the PV curve before (PV 1) would underestimate the stiffness (see Fig. B.14).
